# 162. The Impact of the COVID-19 Pandemic on Antibiotic Prescribing in Pediatric Primary Care

**DOI:** 10.1093/ofid/ofab466.162

**Published:** 2021-12-04

**Authors:** Lauren Dutcher, Yun Li, Giyoung Lee, Robert Grundmeier, Keith W Hamilton, Jeffrey Gerber

**Affiliations:** 1 University of Pennsylvania, Philadelphia, PA; 2 Children’s Hospital of Philadelphia, Philadelphia, Pennsylvania; 3 Hospital of the University of Pennsylvania, Philadelphia, PA

## Abstract

**Background:**

With the onset of the coronavirus disease 2019 (COVID-19) pandemic, pediatric primary care delivery changed rapidly. Prior studies have demonstrated a reduction in ambulatory encounters and antibiotic prescriptions with the pandemic onset; however, the durability of these reductions in pediatric primary care in the United States has not been assessed.

**Methods:**

We conducted a retrospective cohort study to assess the impact of the COVID-19 pandemic and associated public health measures (e.g. social distancing, masking, school closures, and increased availability of telemedicine) on antibiotic prescribing and encounter volume in 27 pediatric primary care practices, and the duration of these changes. Patients under age 19 with an encounter from January 1, 2018 through December 31, 2020 were included. The primary outcome was monthly antibiotic prescriptions per 1000 patients, in the overall population and a subset of encounters with infectious diagnoses, including respiratory tract infections (RTIs). Interrupted time series (ITS) analysis was performed.

**Results:**

There were 60,562 total antibiotic prescriptions from April to December in 2019 and 14,605 antibiotic prescriptions during the same months in 2020, a 76% reduction. The reduction in RTI encounter prescriptions accounted for 91.5% of the overall reduction in prescriptions from 2019 to 2020. Using ITS analysis, there was an immediate decrease from 31.6 to 7.4 prescriptions/1000 patients (predicted means) in April 2020 (-24.2 prescriptions/1000 patients; 95% CI: -31.9, -16.4) (Figures 1 and 2). This was followed by a stable rate of antibiotic prescriptions that remained flat through December 2020. For RTI encounters, a similar pattern was seen, with a decrease by 21.8 prescriptions/1000 patients; 95% CI: -29.5, -14.2) (Figures 1 and 2). Encounter volume also decreased immediately, and while overall encounter volume began returning to a pre-pandemic baseline volume toward the end of the study period, RTI encounter volume remained persistently lower through December 2020 (Figure 3).

Figure 1. Antibiotic prescriptions per 1000 patients prescribed by month from January 2018 to December 2020, overall and for disease-specific subgroups

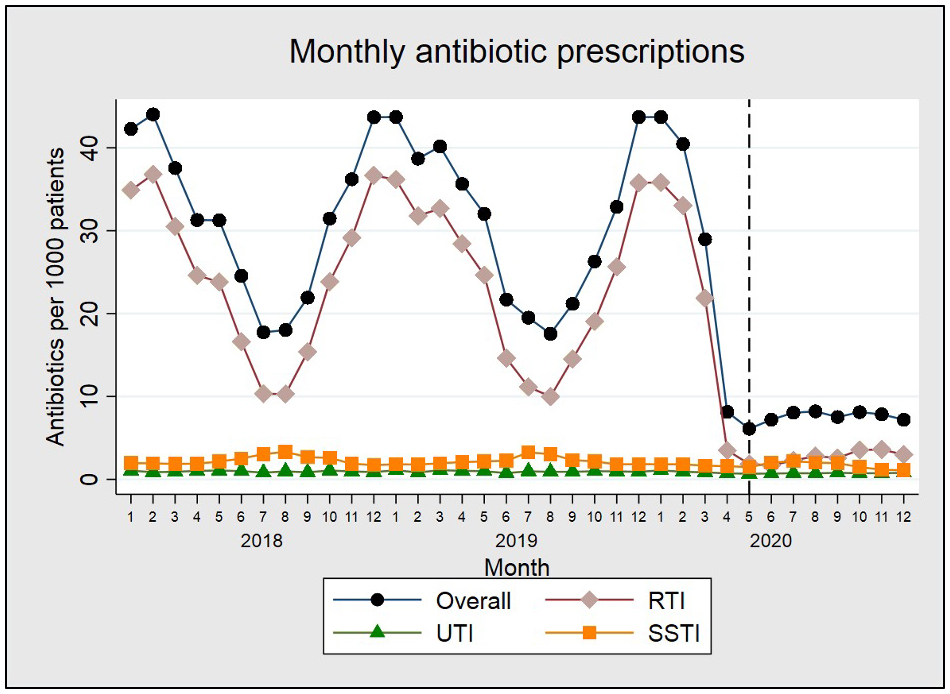

RTI = respiratory tract infection; UTI = urinary tract infection; SSTI = skin and soft tissue infection. Months are numbered sequentially, starting with January (number 1). Dashed line indicates first full month of the pandemic, April 2020.

Interrupted time series analysis for antibiotic prescriptions per 1000 patients by month from January 2018 to December 2020 for (A) all antibiotics as well as antibiotics prescribed at encounters with (B) respiratory tract infections (RTIs), (C) urinary tract infections (UTIs), and (D) skin and soft tissue infections (SSTIs)

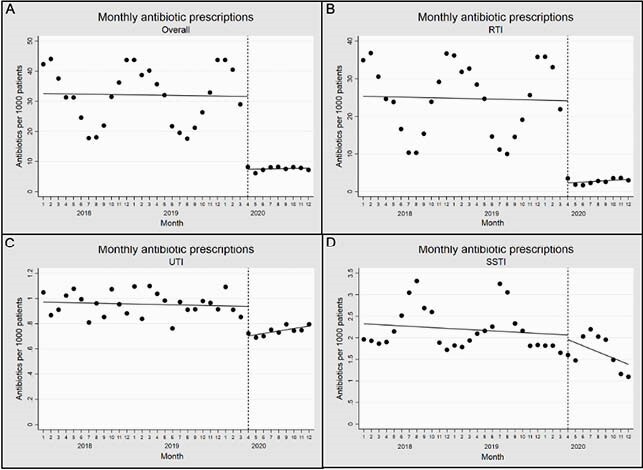

Intervention starts in April 2020 (dashed line). Months are numbered sequentially, starting with January (number 1). Dashed line indicates first full month of the pandemic, April 2020.

Antibiotic prescriptions per 1000 billed encounters by month from January 2018 to December 2020 for (A) all encounters, as well as antibiotics prescribed at encounters with (B) respiratory tract infections (RTIs), (C) urinary tract infections (UTIs), and (D) skin and soft tissue infections (SSTIs)

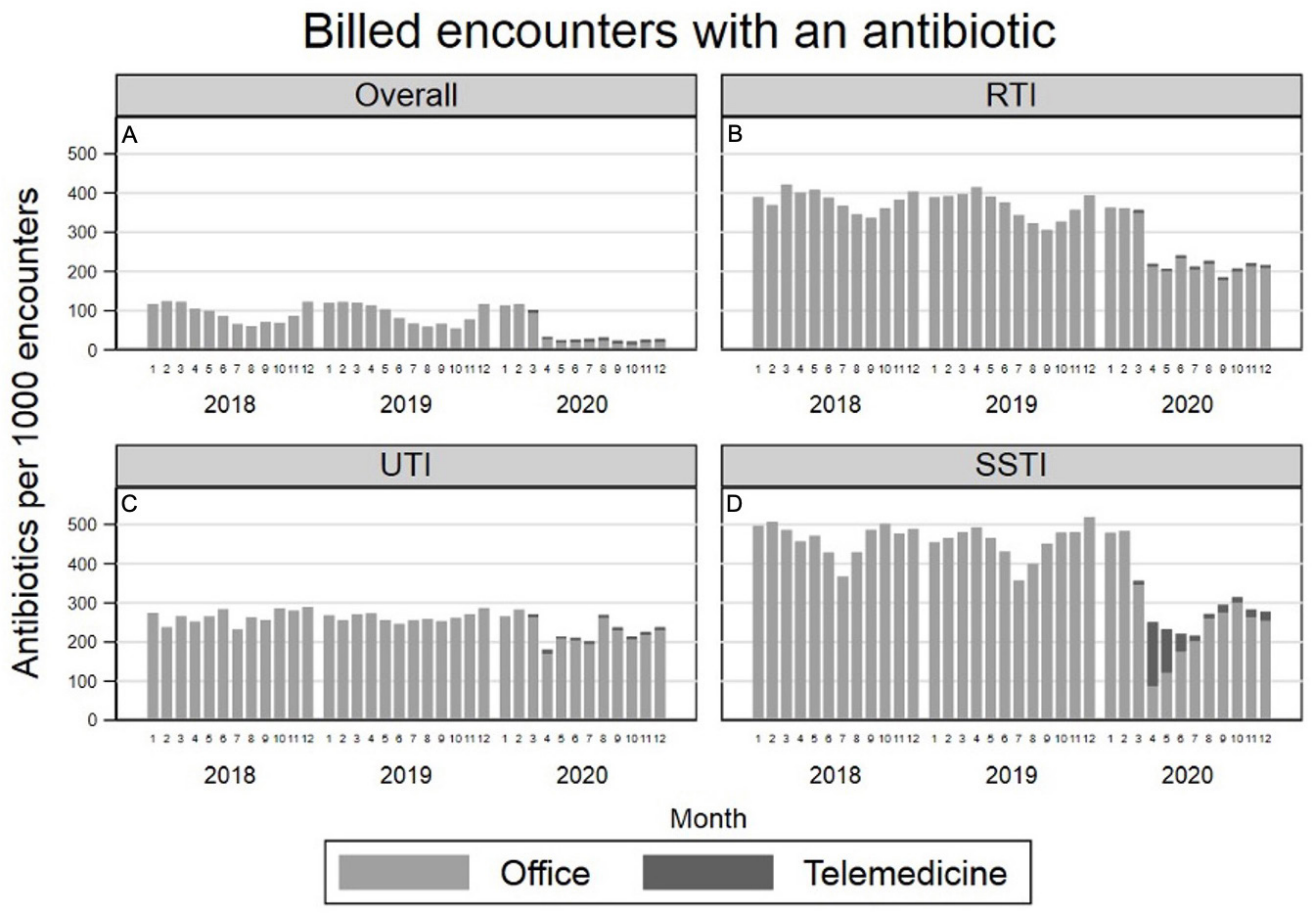

Months are numbered sequentially, starting with January (number 1).

**Conclusion:**

Dramatic reductions in antibiotic prescribing in pediatric primary care during the COVID-19 pandemic were sustained through 2020, primarily driven by reductions in RTI encounters.

**Disclosures:**

**All Authors**: No reported disclosures

